# Menthol cigarettes and the public health standard: a systematic review

**DOI:** 10.1186/s12889-017-4987-z

**Published:** 2017-12-29

**Authors:** Andrea C. Villanti, Lauren K. Collins, Raymond S. Niaura, Stacey Y. Gagosian, David B. Abrams

**Affiliations:** 1 The Schroeder Institute for Tobacco Research and Policy Studies at Truth Initiative, Washington, DC, USA; 2 Vermont Center on Behavior and Health, Department of Psychiatry, University of Vermont, Burlington, VT USA; 3 Department of Health, Behavior and Society, Johns Hopkins Bloomberg School of Public Health, Baltimore, MD USA; 4 Department of Oncology, Georgetown University Medical Center, Lombardi Comprehensive Cancer Center, Washington, DC, USA; 5 Public Policy, Truth Initiative, Washington, DC, USA

**Keywords:** Cessation, Dependence, Policy, Youth tobacco use, Public health

## Abstract

**Background:**

Although menthol was not banned under the Tobacco Control Act, the law made it clear that this did not prevent the Food and Drug Administration from issuing a product standard to ban menthol to protect public health. The purpose of this review was to update the evidence synthesis regarding the role of menthol in initiation, dependence and cessation.

**Methods:**

A systematic review of the peer-reviewed literature on menthol cigarettes via a PubMed search through May 9, 2017. The National Cancer Institute’s Bibliography of Literature on Menthol and Tobacco and the FDA’s 2011 report and 2013 addendum were reviewed for additional publications. Included articles addressing initiation, dependence, and cessation were synthesized based on study design and quality, consistency of evidence across populations and over time, coherence of findings across studies, and plausibility of the findings.

**Results:**

Eighty-two studies on menthol cigarette initiation (*n* = 46), dependence (*n* = 14), and cessation (*n* = 34) were included. Large, representative studies show an association between menthol and youth smoking that is consistent in magnitude and direction. One longitudinal and eight cross-sectional studies demonstrate that menthol smokers report increased nicotine dependence compared to non-menthol smokers. Ten studies support the temporal relationship between menthol and reduced smoking cessation, as they measure cessation success at follow-up.

**Conclusions:**

The strength and consistency of the associations in these studies support that the removal of menthol from cigarettes is likely to reduce youth smoking initiation, improve smoking cessation outcomes in adult smokers, and in turn, benefit public health.

**Electronic supplementary material:**

The online version of this article (10.1186/s12889-017-4987-z) contains supplementary material, which is available to authorized users.

## Background

Menthol has been added to tobacco products as a characterizing flavor since at least the 1920s, but many of the current menthol brands were introduced in the mid-1950s [1, 2]. In 2013, the most recent year of data from the Federal Trade Commission, menthol cigarettes represented 30% of the cigarette market [3]. Tobacco companies have also noted that the menthol segment of the market continues to grow [4], including Reynolds American and Philip Morris USA who have continued to expand their distribution of menthol cigarettes in the past year [5].

The Tobacco Control Act banned all candy and fruit flavors as characterizing flavors of cigarettes. The law did not include menthol in that ban, nor did it address flavors in non-cigarette tobacco products [6]. However, the Act makes clear that the Food and Drug Administration (FDA) has the authority to issue a product standard to ban menthol in cigarettes, or any other tobacco product, to protect public health. In fact, the Act required the Tobacco Product Scientific Advisory Committee (TPSAC), as its first order of business, to review the state of the science on menthol and make a recommendation to the FDA based on the public health standard [7]. TPSAC undertook a review of the science and issued a comprehensive report concluding that it would be in the interest of public health to remove menthol cigarettes from the market [8]. Further, FDA, conducted an independent review of the science in 2013. This report concluded that it is “likely that menthol cigarettes pose a public health risk above that seen with non-menthol cigarettes” [9].

The purpose of the current review was to update the state of the evidence on menthol in cigarettes with respect to two of the three key elements of the public health standard: first, whether there is an increased or decreased likelihood that those who do not currently use tobacco products, most notably youth, will start to use tobacco products; and second, whether there is an increased or decreased likelihood that existing users of tobacco products will stop using such products [10]. In addition to providing a third independent summary of the evidence on menthol, this study highlights findings published after the FDA’s 2013 review.

## Methods

We undertook a systematic review using a PubMed search of the peer-reviewed literature through May 9, 2017 with the terms “menthol AND cigarette*.” The National Cancer Institute’s Bibliography of Literature on Menthol and Tobacco [11] and the FDA’s original 2011 report [9] and 2013 addendum [12] were reviewed for additional publications not captured in the PubMed search. Articles published prior to 2013 were reviewed for inclusion and coded by AV; articles published after 2013 were reviewed for inclusion by LC and coded by LC and AV. In 2016, the review was moved into a centralized database and searches were rerun within Eppi-Reviewer 4 (EPPI-Centre, University of London); at this time, all abstracts were double-checked against the inclusion criteria for quality control purposes. The May 2017 search update was conducted within the Eppi-Reviewer platform. Lab-based studies and studies with no direct comparison between menthol and non-menthol use were excluded. Published reviews, commentaries, case reports, editorials, letters to the editor, meeting proceedings, and policy statements were also excluded. Included studies were classified into at least one of 6 categories, including 1) Initiation; 2) Dependence; 3) Cessation; 4) Prevalence; 5) Marketing; and 6) Policies.

Since the main goal of the current review was to update a narrative review on the Initiation, Dependence, and Cessation categories and a range of study types were included, we did not employ a standardized assessment of the quality of included studies (e.g., PRISMA checklist). To synthesize the evidence for these three categories, we:

1. Examined the methods and designs of the studies, the rigor with which they were conducted, and the limits of interpreting data with respect to the population, place, and time of the study;
2. Categorized individual studies according to their methods and design and evaluated studies that used comparable methods to determine consistency of the evidence across populations and over time. We examined evidence across these comparable studies to assess the strength of the association and to determine if a temporal relationship was present between menthol cigarette use and smoking initiation or cessation;
3. Evaluated the body of scientific evidence to determine whether findings of individual studies were coherent with each other and with our broader understanding of tobacco use in the United States; and
4. Considered the plausibility of these findings in the context of tobacco industry and related documents.

Finally, we asked whether positive associations exist and whether chance, bias, and confounding could be ruled out with reasonable confidence. In keeping with a classification scheme based on FDA’s public health standard, and recognizing that decision-makers must often act in the face of scientific uncertainty, we asked whether the evidence in a particular area was sufficient to conclude that a relationship was more likely than not, whether the evidence shows that a relationship was at least as likely as not, whether the evidence is insufficient to conclude that a relationship was more likely than not, or whether there was insufficient evidence to make a determination of strength of evidence. The focus of the evidence synthesis was on studies conducted in the United States; data presented from other countries is noted as such throughout the text.

## Results

**Fig. 1:**
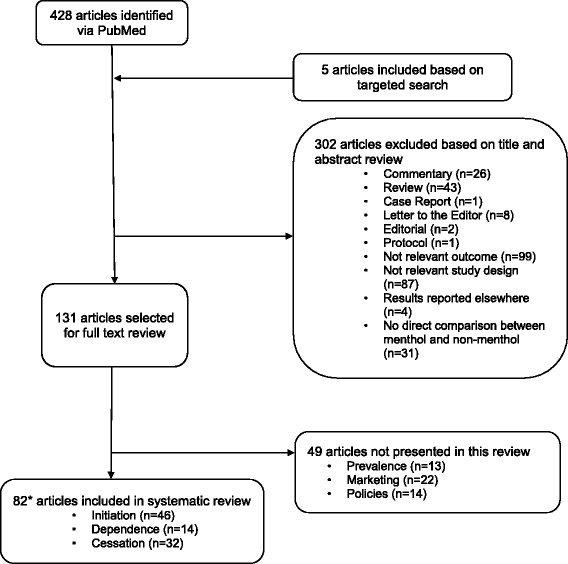
Flowchart of studies included in the menthol systematic review

Of the 131 empirical articles on menthol cigarettes included in the full review (see Fig. 1), 82 were relevant to initiation (*n* = 46; Additional file 1: Table S1), dependence (*n* = 14; Additional file 2: Table S2), and cessation (*n* = 34; Additional file 3: Table S3). The remaining 49 articles addressed other topics: prevalence (*n* = 13), marketing (*n* = 22), and policies (*n* = 14). Thirty-three of these articles were published after 2013. Details on the findings by study category are described in detail below.

### Initiation

#### The prevalence of menthol cigarette use is higher in youth than young adults and adults

A 2015 study using 2004–2010 data from the National Survey on Drug Use and Health (NSDUH), adjusted for misclassification of menthol brand, showed that from 2008 to 10, 56.7% of youth smokers (aged 12–17) smoked menthol cigarettes [13]. This compares with an overall menthol cigarette prevalence (youth and adults) of 35.2% and represents 1.2 million menthol smoking youth. A 2016 follow-up study in NSDUH highlighted that the percentage of menthol cigarette smokers increased 4.1 percentage points between 2008–2010 and 2012–2014, with youth smokers remaining the age group with the highest prevalence of menthol cigarette use [14]. These findings were also confirmed using 2013–2014 data from the Population Assessment of Tobacco and Health (PATH) Study [15]. Among current cigarette smokers, 59.5% of youth used mentholated cigarettes compared to 37.1% of adults. When looking only at exclusive cigarette smokers, the prevalence of mentholated cigarette use remained higher in youth (56.5%) compared to adults (39.5%).

Black smokers report a high prevalence of menthol cigarette use, regardless of age [13, 16–21]. A cross-sectional study of adult daily smokers found that nearly 80% of black smokers smoked menthol cigarettes, the highest prevalence across racial/ethnic groups [22]. Controlling for gender, race/ethnicity, household income and days smoked in the past month, the odds of smoking mentholated brands were more than threefold higher in the youngest age groups (12–15 and 16–17) of smokers compared to smokers aged 35 and older in both 2008–2010 [13] and 2012–2014 [14]. These estimates are slightly higher than those published in the 2009 *NSDUH Report: Use of Menthol Cigarettes* [16] and NSDUH analyses by Caraballo and Asman [19] and Rock et al. [18], but account for two more years of data collection and adjustment for misclassification of menthol status. Together, these studies demonstrate the stability of these nationally-representative estimates over seven years highlighting higher rates of menthol use in youth compared to adults from 2004 to 2014.

#### There is a persistent age gradient in menthol cigarette use among the youngest smokers

Results from the 1999, 2000, and 2002 National Youth Tobacco Survey (NYTS), a survey administered to approximately 25,000 middle and high school students in each wave, confirm a statistically significantly higher prevalence of menthol cigarette use among middle school students compared to high school students [23–25]. Results differ for some racial/ethnic subgroups [26, 27]. In the 2006 NYTS, 57.1% of middle school smokers reported that their usual brand was menthol compared to 43.1% of high school smokers [28]. Data combined for years 2004, 2006, and 2009 of the NYTS showed that 49.4% of middle school current smokers reported smoking menthol cigarettes compared to 44.9% of high school current smokers [19]. In 2004 and 2006 NYTS, Newport was the second most popular brand among youth smokers [29].

Studies of youth and adults published prior to 2013 highlight that the highest prevalence of menthol cigarette use occurs among youth smokers, followed by young adult smokers, and that both are significantly higher than menthol cigarette use among older adult smokers [17–19]. These findings are consistent with studies using more recent data that were published after 2013 [13–15, 30].

Other recent national studies examining adults only consistently report that young adult smokers (aged 18–24 or 18–25) are significantly more likely to use menthol cigarettes than older adult smokers (aged 25+ or 26+), even after controlling for other potential confounders including socioeconomic status, sexual orientation [31], and psychological distress [32]. One study in a national sample of young adults aged 18–34 found that menthol cigarette smokers were significantly younger than non-menthol cigarette smokers in bivariate analyses, but this did not persist in multivariable models, likely due to the restricted age range of the sample [33].

#### Menthol cigarette use among youth has not decreased in the past decade, despite decreases in non-menthol cigarette use

Giovino et al. showed that the prevalence of smoking menthol cigarettes remained constant among youth (aged 12–17) from 2004 to 2010, at the same time that the prevalence of non-menthol cigarette use decreased significantly in this age group [13]. Furthermore, menthol cigarette use significantly increased over this time period in young adults (aged 18–25) while the prevalence of non-menthol cigarette use decreased significantly. These findings were consistent with the 2011 NSDUH report on *Recent Trends in Menthol Cigarette Use* [17]. In updated NSDUH data from 2014, menthol cigarette prevalence was higher than non-menthol cigarette prevalence in youth and young adults [14].

#### Recent youth initiates are significantly more likely to use menthol cigarettes than youth who have smoked longer than one year

Estimates from the NYTS and NSDUH also demonstrate increased menthol cigarette use among recent youth initiates. Two studies [16, 34] combining waves of national data on youth smoking report a higher prevalence of menthol cigarette use among youth who have been smoking less than one year compared to those who have smoked more than one year. One of the studies combined data from five years of the NSDUH (2004–2008) and the other used two years of data from the NYTS (2000 and 2002). In the NSDUH study, past month smoking of menthol cigarettes was more likely among smokers aged 12–17 who began smoking in the past 12 months than among those who had been smoking for more than a year (49.2% vs. 43.8%); findings were similar in young adults where past-year initiates had higher menthol use than longer-term smokers (40.2% vs. 36.4%) [16]. The 2011 NSDUH report on menthol also reported that the prevalence of menthol use in recent initiates among all participants aged 12+ increased during 2007–2010 as compared to 2004–2006 and that past month menthol use was higher among recent initiates compared to longer-term smokers in both time periods [17]. In the NYTS study, middle school students who had been smoking for less than 1 year were significantly more likely to smoke menthol cigarettes compared with middle school students who had been smoking for more than 1 year (62.4% vs. 53.3%, *p* = 0.002) [34]. Two recent analyses in the NYTS data [19, 28] did not find a significant relationship between menthol cigarette use and smoking initiation among adolescents. One study using 2006 NYTS data shows that the proportion of middle school smokers whose usual brand was menthol was higher among those who smoked for 1 year or more (54.7%) than among those who smoked for less than a year (42.2%) [28]. Among high school youth, these percentages were similar for smokers who had smoked for less than and for more than 1 year (42.8% vs. 43.1%). Another study combining data across years of the NYTS (2004, 2006, and 2009) used cigarettes smoked per day and days smoked per month as proxy measures for early “stages” of use (initiation) and showed no difference in the prevalence of menthol use by “stage” [19].

#### Longitudinal studies demonstrate that initiation with menthol cigarettes facilitates progression to established use in young smokers

Prior to 2014, one cross-sectional study and two longitudinal studies assessed the impact of menthol initiation on smoking behavior. Conducted in a southeastern city, the cross-sectional study showed that black middle and high school students, who smoke at lower rates than whites, greatly accelerate their cigarette consumption when their brand of choice contains menthol [35]. African American menthol users were between 1.7 and 3.5 more likely to fall into a higher category of cigarette consumption than whites. A longitudinal study, conducted by Nonnemaker et al. [36], documents that adolescents who initiated smoking with menthol cigarettes during the course of a cohort study were more likely to progress to established smoking by the end of the three-year study compared to those who initiated with non-menthol cigarettes. The stringency of the definition of “established smoking” in this study (i.e., at least 100 cigarettes lifetime plus smoking on 20–30 of the past 30 days) provides strong evidence for the relationship between menthol cigarette use and progression to regular use given the typical adolescent definition of current cigarette use as any use in the past 30 days. The second longitudinal study, published by Dauphinee et al. [37] shows that recognition of Newport cigarettes, a leading menthol brand, was associated with smoking experimentation in a large sample of adolescent never-smokers at 12-month follow-up.

Findings from four recent cross-sectional studies further support these findings. One cross-sectional study of a nationally-representative sample of Canadian high school students showed that menthol smoking youth had a significantly higher odds of reporting intent to continue smoking compared to non-menthol smoking youth [38]. These findings held when examining established and experimental smokers separately. A second cross-sectional study examined changes in smoking behavior using a national sample of young adult smokers and showed that menthol cigarette use nearly doubled the odds of increased smoking behavior, including transitioning from no smoking to current smoking or from someday to every day smoking in the past year [39]. These findings are consistent with recent analyses in Wave 1 of the PATH study that documented a strong association between first use of a flavored tobacco product and current tobacco use among youth and adults [15]. A fourth cross-sectional study, which conducted regression analyses using data from four nationally representative samples of youth and adult current smokers, found that current menthol use was not associated with an increased odds of being a daily versus non-daily smoker in youth and adults [40].

#### Young smokers are likely to remain with their “starter” type of cigarette over time

Data from the National Youth Smoking Cessation Survey (NYSCS), a two-year (2003–2005) longitudinal telephone study of adolescent and young adult cigarette smokers aged 16–24 confirm that 85% of baseline menthol smokers remained menthol smokers at 24 months and 93% of baseline non-menthol smokers remained non-menthol smokers [41]. In a study published in 2013 by Nonnemaker et al., the majority of adolescent smokers who initiated with menthol cigarettes remained menthol smokers at follow-up (63%); this was similar to the proportion of adolescent smokers who initiated with non-menthol cigarettes and remained with non-menthol smokers at follow-up (62%) [36].

Two studies published after 2013 support these findings. One study, conducted over one year in the Truth Initiative Young Adult Cohort, bolsters the findings that the majority of young adult smokers, aged 18–34, remain with their initial type of cigarette over time [42]. In this study, young adults smokers who initiated with menthol cigarettes were more than eight times more likely to remain menthol cigarette smokers than those who initiated with non-menthol cigarettes. The second study, focused more broadly on flavored tobacco use in Wave 1 of the PATH study, found first use of a flavored tobacco product was associated with a more than two-fold higher prevalence of exclusive menthol cigarette use in adults, with young adults being more likely to use menthol cigarettes [15].

#### The findings regarding an age gradient in menthol cigarette use – Increased levels of menthol smoking in the youngest age groups -- are not attributable to menthol brand misclassification or socioeconomic status

Misclassification of menthol cigarette use has been identified in youth studies [28] and tobacco control researchers have also raised the notion that menthol cigarette use may be associated with economic pressure to use fewer cigarettes [43], thus menthol use may be due to lower socioeconomic status. These data show that the age gradient in use is not an artifact of misclassification of menthol use [23]. They also highlight that use of menthol cigarettes is not explained by socioeconomic status, assessed as household income.

Four papers published after 2013 confirm these earlier results. Analyses using 2008–2009 NSDUH data support that young adults (aged 18–25) are significantly more likely to use menthol cigarettes than older adults, after controlling for age, gender, race/ethnicity, education, income, marital status, health insurance, cigarettes per day, time to first cigarette, and psychological distress [32]. Giovino et al. addressed potential misclassification of menthol brand among youth and adults in 2008–2010 NSDUH data, showing a persistent age gradient in menthol cigarette use across gender, race/ethnicity, household income, and number of days smoked per month [13]. These findings held in updated analyses of 2012–2014 NSDUH data [14]. A fourth study published in 2016 using 2012–2013 NSDUH data showed that menthol cigarette use was also not explained by urban/rural differences [44].

#### Menthol cigarette smoking is correlated with other risk behaviors in young people

Menthol cigarette smoking has been associated with other tobacco use in young adults (small cigars [45] and other flavored tobacco products [46]) and alcohol and marijuana use in youth [47–49]. In a community-based sample of adolescents in the U.S., past 30-day menthol cigarette smokers reported higher lifetime marijuana use, but not marijuana use in the past 30 days compared to non-menthol smokers [48]. In a sample of adolescent daily smokers seeking cessation treatment, menthol cigarette use was correlated with past 30-day marijuana use [48].

In a nationally-representative sample of Canadian 7th through 12th grade students published after 2013, menthol cigarette smokers were significantly more likely to report binge drinking or using marijuana in the past year compared to non-menthol smokers [47]. In national NSDUH data collected in 2013 and 2014 among participants aged 12 and older, a higher percent of marijuana/menthol cigarette users were 12–17 years of age compared to other usage groups (i.e., marijuana/non-menthol cigarettes, menthol cigarettes only, non-menthol cigarettes only) [49].

#### The tobacco industry has long understood the appeal of menthol cigarettes as starter products for youth

Historical tobacco industry documents underscore menthol brands as starter products for youth (i.e., “Menthol brands have been said to be good starter products because new smokers appear to know that menthol covers up some of the tobacco taste and they already know what menthol tastes like, vis-à-vis candy” [50]) and recognize the importance of adolescent smokers to the success of menthol brands (i.e., “The success of Newport has been fantastic during the past few years. Our profile taken locally shows this brand being purchased by black people (all ages), young adults (usually college age), but the base of our business is the high school student” [51]). Recent tobacco industry document reviews have also underscored the relationship between menthol cigarette use, youth smoking initiation and tobacco dependence, as understood and manipulated by the tobacco industry [52–54]. Data from financial analysts support that the menthol marketplace is strongly influenced by youth smoking. Tobacco industry experts at Morgan Stanley noted in 2012 that menthol cigarettes continue to have a higher market share in younger age groups, despite the fact that youth smoking continues to decline [55]. Increased market share of menthol cigarettes among youth has also been documented outside the U.S. [56, 57].

In two studies published after 2013, the appeal of menthol flavoring was demonstrated to influence intention to smoke and initial smoking [58, 59].

#### Summary - initiation

Fifteen years of national studies of tobacco use across different populations and time periods arrive at the same conclusions: there is a strong pattern of a higher – and growing – proportion of menthol cigarette use among youth (aged 12–17) than adults, and especially among younger adolescents and recent youth initiates. The results from large, representative studies provide evidence of an association between menthol and youth smoking that is robust and consistent in magnitude and direction and is unlikely to be due to bias, confounding, or chance. Among all youth and young adults, not just current smokers, the prevalence of smoking non-mentholated brands decreased from 2004 to 2014; as of 2014, menthol cigarettes were more prevalent than non-menthol cigarettes in youth and young adults, indicating that menthol cigarettes are gaining market share in these age groups.

More particularly, the replication of these findings over time using different studies and populations provides evidence of consistency. Data showing a high prevalence of menthol use among youth, in addition to higher prevalence among younger adolescents and recent initiates, and stable or increasing menthol cigarette use over time – despite reductions in non-menthol cigarette use – supports coherence of the evidence on menthol and youth smoking. Plausibility of the relationship between menthol and youth smoking is corroborated by historic industry and related documents on the development and marketing of mentholated cigarettes to youth [50, 51]. The magnitude and statistical significance of the data on the increasing proportion of menthol use and brand preference among youth over time reveals that this is a national phenomenon. Additional analyses exclude misclassification and socioeconomic status as explanations for the high prevalence of menthol cigarette use among youth.

### Dependence

#### Youth menthol smokers report greater levels of nicotine dependence than youth non-menthol smokers

Of eight studies assessing nicotine dependence among youth [28, 34, 36, 60–64], five demonstrate significantly higher endorsement of dependence symptoms among menthol smokers compared to non-menthol smokers [28, 34, 36, 60, 62]. Of the three studies using NYTS data from 2000, 2002, 2004, and 2006, two [28, 62] report that young menthol cigarette users have a significantly shorter first time-to-cigarette after waking, which is a hallmark of nicotine dependence [65], after adjusting for gender, race, grade, number of days smoked in the past 30 days and number of cigarettes smoked per day. These two studies also show greater endorsement of withdrawal symptoms among youth menthol smokers, particularly, craving [28, 62], and feeling irritable or restless after not smoking for a few hours [28]; these findings also adjusted for gender, race, grade, number of days smoked in the past 30 days and number of cigarettes smoked per day. This is consistent with the third NYTS paper that highlights higher than median scores on a nicotine dependence scale among youth menthol compared to non-menthol smokers, controlling for age, gender, race/ethnicity, and smoking behavior (i.e., length, frequency, and level of smoking) [34]. A smaller cross-sectional study of adolescents recruited for a cessation treatment study by Collins and Moolchan also reported a greater proportion of adolescent menthol smokers smoking within five minutes of waking compared to non-menthol smokers [60]. Further, a national longitudinal study of U.S. adolescents reported that initiating smoking with menthol cigarettes was associated with higher nicotine dependence score, controlling for gender, age, race/ethnicity [36]. Two of the remaining three studies showed no differences in adolescent nicotine dependence in menthol versus non-menthol smokers using the Hooked on Nicotine Checklist [61, 63]. The third study, which used data from four nationally representative samples of youth and adults, found that menthol smokers do not report a higher Heaviness of Smoking Index, compared to non-menthol smokers [64].

#### Adult menthol smokers report shorter time to first cigarette than non-menthol smokers

Six studies in adults also focus on nicotine dependence among menthol compared to non-menthol smokers by assessing time to first cigarette [6, 66–70]. Two studies in women show that female menthol smokers have a significantly shorter time to first cigarette than non-menthol smokers [66, 68]. A study in a sample of current daily smokers from 1990 to 2001 reported a significantly shorter time to first cigarette among Black menthol users compared to non-menthol users, but this relationship was not present among White smokers [67].

Two studies in adult current smokers published after 2013 found no significant difference in time to first cigarette between menthol and non-menthol cigarette smokers [69, 70]. However, one other study was more aligned with earlier findings. The study of adult daily smokers found that menthol smokers were significantly more likely to report that they would hate to give up the first cigarette in the morning more than any other compared to non-menthol smokers [6].

#### Summary - dependence

Of fourteen studies published over a fifteen-year period, nine show that menthol smokers report increased nicotine dependence compared to non-menthol smokers [6, 28, 34, 36, 60, 62, 66–68]. The data on dependence among youth menthol smokers are particularly strong, given that four [28, 34, 36, 62] of the five studies showing an association control for a number of important confounders and one of these documents a temporal relationship between initiation with menthol cigarettes and the subsequent development of a higher level of nicotine dependence compared to initiation with a non-menthol cigarette [36]. All six of the studies in adults are cross-sectional, of which four demonstrate a shorter time-to-first cigarette among menthol smokers compared to non-menthol smokers. Three of these four studies examine women [66, 68] and Blacks [67], both groups targeted by tobacco industry marketing [71].

The findings on increased nicotine dependence among youth and adults are particularly important because they highlight a potential mechanism linking experimentation with cigarettes through progression to regular use, and subsequently, reduced cessation among menthol smokers. As a result, it is very likely that a ban on menthol in cigarettes would reduce nicotine dependence at the population level, thus having tremendous impacts on both initiation and cessation of cigarette use.

### Cessation

In examining evidence on the relationship between menthol cigarette use and smoking cessation, we focused on studies that used cessation measures in addition to measures of quit attempts or intention to quit; as a result, there are several studies using intention to quit or quit attempts as the primary outcome that are not addressed in detail in this section [42, 72–74].

#### National cross-sectional studies

Five studies in the Tobacco Use Supplement to the Current Population Survey (TUS-CPS) measure cessation outcomes beyond quit attempts or intention to quit. Three studies [75–77] demonstrate that menthol users are less successful in quitting than non-menthol users despite increased quit attempts or intentions to quit. One of these studies found that past-year quit attempts were significantly increased in menthol compared to non-menthol smokers, but short-term (greater than 3 months and less than one year) and longer-term (greater than 3 months and less than five years) quit rates were significantly lower among those who smoke menthol cigarettes as compared to non-menthol cigarettes [75]. One study exploring cessation by race/ethnicity reported that non-Hispanic white, African American, and Puerto Rican menthol smokers were less likely to have quit smoking in the past five years compared to their non-menthol smoking counterparts [76]. Another study examining cessation by racial/ethnic groups found that cessation of at least six months was significantly reduced by 52% to 78% in African American, Hispanic/Latino, Asian American/Pacific Islander, and non-Hispanic white menthol smokers compared to non-menthol smokers [77]. Two studies found no difference in cessation outcomes among menthol and non-menthol smokers [78, 79]. One study examined quitting behaviors among daily menthol and non-menthol smokers with similar cigarette consumption patterns and found no difference in quit attempts or greater than two-week abstinence by menthol status [78]. One study published after 2013 among current and past-year smokers (recent active smokers) found no difference in quit intention, quit attempts, or quit rate among menthol compared to non-menthol smokers [79].

Studies of adult smokers in the 2005 National Health Interview Survey (NHIS) Cancer Control Supplement corroborate the findings for reduced cessation among racial and ethnic subgroups from the TUS-CPS data. These studies report increased quit attempts in the past year among menthol compared to non-menthol smokers [80, 81] but significantly reduced cessation among African-American [80, 82] and Hispanic menthol smokers compared to non-menthol smokers [82]. One of these studies [82] also collapsed Hispanic and African-American smokers into one category and reported a statistically significant decrease of 45% in the odds of cessation among non-White menthol smokers compared to non-White non-menthol smokers. One study assessing quit duration as a cessation measure showed that there was a significant increase in quit duration among white female menthol smokers compared to white female non-menthol smokers, but no statistically significant differences among the other five demographic groups [81].

A more recent study examined the association between menthol use and the likelihood of being a former versus current smoker using data from the TUS-CPS (2010/11) and the NHIS (2005 and 2010). Analyses of the TUS-CPS found a statistically significant inverse association between menthol use and having quit smoking, but this was not reported when using the NHIS [83].

#### Community-based studies

One study from 1981 to 1999 in a hospital-based study of 19,545 current and former smokers showed that Black and White menthol users were significantly less likely to be former smokers compared to non-menthol users, but was no longer significant after controlling for age, sex, education, case–control status, years of smoking, and cigarettes per day [84]. Another study of 480 inner-city adult current smokers reported that menthol smokers reported a more recent quit attempt compared to non-menthol smokers (12 vs. 24 days; *p* = 0.047), but there was no difference in most recent or longest ever duration of abstinence [85]. A third study of 928 female smokers screened for a smoking cessation study reported that fewer menthol smokers reported a previous quit attempt of greater than 90 days compared to non-menthol smokers [68]. In a hospital-based study of 1067 adult smokers there was no significant effect of menthol use on motivation to quit and confidence to quit when adjusting for age, sex, race, income, education, and tobacco dependence [86].

#### Cohort studies

Of eight cohort studies examining differences in smoking cessation [87–94], three reported significantly lower quit rates among menthol smokers compared to non-menthol smokers at follow-up [90, 91, 94]. The study by Pletcher et al. [90] showed a 37% reduction in the odds of sustained cessation adjusted for age, sex, and ethnicity, but this result did not retain statistical significance after additional adjustment for educational level, marital status, employment, and health insurance status. The second study by Gandhi et al. [91] reported significant reductions in the odds of cessation of 68% and 57% among African American and Latino menthol smokers, respectively, at 4-week follow-up and a decrease of 52% in African Americans at 6-month follow-up, controlling for age in years, education, gender, employment status, type of insurance, cigarettes per day, age smoked for first time, awaken at night to smoke, time to use first cigarette of day, previous attempts to quit smoking, and the presence of a disease caused or aggravated by smoking. The third study published in 2014 by Lewis et al. [94] found menthol smokers to be less likely to quit (17.1% in African Americans, 24.2% in non-African Americans) than non-menthol smokers (21.9% in African Americans, 29.4% in non-African Americans).

Two additional studies by Reitzel et al. showed significant reductions in cessation in White menthol smokers, adjusted for covariates including age, partner status, income, and education; one for long-term (approximately 6 months) continuous abstinence in pregnant smokers [87] and a more recent publication for short-term abstinence in adult daily smokers [93]. Three other studies did not show a difference in abstinence at follow-up in menthol compared to non-menthol smokers [88, 89, 92]. The COMMIT study [89], which did not show a difference in cessation between menthol and non-menthol smokers, surveyed smokers in selected communities in the U.S. and Canada between 1988 and 1993. Possible reasons for the mixed results across the three studies include population sampling and recentness of the data.

Of the five studies showing a statistically significant difference in cessation by menthol smoking status, one [91] was conducted in a cessation clinic population from 2001 to 2005, one [90] in a large cohort of healthy young African American and European American men and women in four US cities from 1985 through 2000, one [94] in a sample of nationally representative U.S. households from 2004 to 2009, and two others in community-based samples in Houston, Texas between 2004 and 2008 [87, 93]. The two other studies showing no effect of menthol on cessation were conducted in southern States from 2002 to 2009 [92] and in Minnesota between 2009 and 2011 [88]. We would note that the cigarette market has undergone dramatic changes over the past 10–15 years, including the introduction of a number of new menthol brands. Because of the differences in menthol levels and effects among brands [95], it is important to rely on the most recent data that reflects products currently on the market. Accordingly, we consider the COMMIT study less relevant to the question of adult cessation in the context of an FDA ban on menthol, as it includes older data. Additional weight should also be given to the cohort study conducted in a cessation clinic [91], as it reflects smokers who are motivated to quit and thus, controls for confounding by cessation cognitions and intention to quit.

#### Randomized controlled trials

Seven randomized controlled trials [96–102] in populations motivated to quit smoking explored the impact of menthol cigarette use on cessation. One study testing the impact of a phone survey and provider progress notes on smoking cessation among VA patients showed no difference six months after the intervention in smokers who had not smoked in the past seven days [96]. An additional study among stimulant-dependent adults found no significant association between cigarette type and smoking cessation [100]. However, five studies [97–99, 101, 102] testing the effect of pharmacotherapies and behavioral therapies on smoking cessation reported significantly reduced cessation among menthol smokers compared to non-menthol smokers. While results in two of these studies [97, 98] maintained a consistent direction (i.e., menthol users had reduced cessation compared to non-menthol users), they were not statistically significant across all follow-up time points; three of these studies reported significantly reduced cessation among menthol smokers at all time points assessed [99, 101, 102]. In the 2003 study by Okuyemi et al. [97], African American menthol smokers had significantly reduced 7-day point prevalence abstinence at 6 weeks (28.3% vs. 41.5%; *p* = 0.006) compared to African American non-menthol smokers, but the difference was not significant at 6 months (21.4% vs. 27.0%; *p* = 0.21). In the 2007 study of African American light smokers (≤ 10 cigarettes per day) by Okuyemi et al. [98], menthol smokers had significantly reduced 7-day point prevalence abstinence at 26 weeks (11.2% vs. 18.8%; *p* = 0.015) compared to non-menthol smokers, but not at 8 weeks (22.6% vs. 26.8%; *p* = 0.291). The 2013 study of African American light smokers by Faseru et al. [99] showed significantly reduced cotinine-verified 7-day point prevalence abstinence among menthol compared to non-menthol smokers at week 7 (14.4% vs. 28.4%; *p* = 0.001) and week 26 (10.0% vs. 20.4%; *p* = 0.005); this study also demonstrated an 84% increased odds of cessation among non-menthol compared to menthol smokers, controlling for treatment, visit attendance, cotinine level, and years smoked. In the 2014 study of treatment–seeking smokers by Rojewski et al., [101] menthol smokers showed significantly reduced 7-day point prevalence abstinence among menthol compared to non-menthol smokers at week 14 (14.8% vs. 33.3%; *p* = 0.04) and week 26 (13% vs. 30%; *p* = 0.04). In the 2014 study by Smith et al. [102], menthol smoking was associated with reduced likelihood of smoking cessation success compared to non-menthol smoking (31% vs. 38%); this study also found that among menthol smokers, African American women were at a particularly high risk of cessation failure compared to white women (17% vs. 35%; OR = 2.63, 95% CI = 1.75,3.96). One major difference in these studies is focus of the cessation intervention.

Five studies [97–99, 101, 102] testing the impact of an individual-level intervention showed reduced cessation among menthol smoking participants while the provider-focused intervention [96] showed no difference in cessation among menthol and non-menthol smoking participants. One individual-level intervention did not show a difference in cessation by menthol use, but that may be attributed to its unique population and the effect of smoking on the participants’ other substance use. The studies focusing on individual-level interventions are more relevant to the question of menthol’s influence on smoking cessation, as they capture a seven to eight-week window of evidence-based treatment for smoking cessation rather than a single provider visit. The five studies of African American [97–99, 102] and treatment-seeking [101] smokers provide particularly strong evidence of reduced cessation among menthol compared to non-menthol smokers in the face of extended smoking cessation treatment.

#### Summary - cessation

Four of five studies in the TUS-CPS [75–77, 83] and two of four studies in the Cancer Control Supplement to the National Health Interview Survey [80, 82] that examined quit attempts and additional cessation measures among adult smokers indicate that cessation is reduced in non-Hispanic whites and in racial and ethnic subgroups of menthol smokers compared to non-menthol smokers despite increased quit attempts. These findings demonstrate reasonable consistency and a coherent picture of quit behavior among menthol smokers: menthol smokers make more quit attempts than non-menthol smokers, yet have a more difficult time quitting successfully. Five [87, 90, 91, 93, 94] of eight cohort studies and five [97–99, 101, 102] of seven randomized controlled trials contribute to the consistency of the findings and the strength of the association between menthol smoking and reduced cessation among adult smokers. Evidence from these ten studies with consistent results also support the temporal relationship between menthol smoking and reduced smoking cessation through their study designs which included longitudinal follow-up of adult smokers. One community-based cross-sectional study also indicates that female menthol smokers have reduced cessation success [68]. One study using consumer purchasing data also shows that African American menthol smokers are less likely to quit smoking [94]. Further, these findings are plausible in light of historic tobacco industry marketing of menthol cigarettes as medicinal, less harmful, or even a more healthful product than non-menthol cigarettes [103–106] and the resulting perceptions among menthol smokers that menthol cigarettes may be less risky than regular cigarettes [107]. These population-based cross-sectional, cohort, and randomized controlled studies, which showed strong and consistent associations between menthol use and reduced smoking cessation, were high quality, and addressed bias and confounding through regression adjustment or randomization.

## Discussion

Studies published after 2013 bolster and augment earlier findings regarding the deleterious relationship between menthol cigarette use, youth smoking initiation, and nicotine dependence. The strength and consistency of the associations in these studies confirm the conclusions of previous studies and provide additional support for the conclusion that an FDA ban on menthol tobacco products would benefit public health.

Limitations of this review include restriction of the search to articles published in PubMed and lack of multiple independent coders which may have biased the way that studies were included and characterized. Additionally, brand names (e.g., Newport) were not included in the search strategy, which may have resulted in not capturing all relevant studies.

Studies of the cigarette marketplace confirm menthol’s growing market share. The proportion of menthol variants of popular brands like Pall Mall, Camel, and Marlboro rose, at times substantially, between 2004 and 2013 [108]. Newport, the leading menthol brand, increased its market share from 7.23% in 2002 to 10.89% in 2013 [108] and has continued to grow following Reynolds American’s 2015 acquisition of Lorillard Tobacco Company [109], from 13% to 13.6% in the fourth quarter of 2015 alone [110]. More recently, Newport launched new promotional efforts aimed at recruiting young adults to smoke cigarettes [111].

Analyses of the NSDUH highlight that among past 30-day smokers, the proportion of menthol cigarette users was 35% in 2008–2010 and increased significantly to 39% in 2012–2014 [14]. These increases were observed in young adults aged 18–25, as well as adults aged 26–34 and 35–49 and over this time period, youth smokers aged 12–17 remained the group with the highest prevalence of menthol cigarette use (54%) [14]. The findings of this review, in concert with recent evidence on the increasing presence of menthol in the cigarette market, underscores the urgent need for policy action to ban the sale, marketing, or presence of menthol as a characterizing flavor in cigarettes at the national, state, and local levels.

## Conclusions

This review of the scientific evidence demonstrates that there is more than sufficient evidence to establish a positive relationship between menthol cigarettes and (1) increased youth smoking initiation, (2) increased nicotine dependence, and (3) decreased adult cessation. The weight of the evidence from studies published through 2017 supports that removal of menthol from cigarettes would, in the words of the Tobacco Control Act, decrease the likelihood that those who do not use tobacco products will start using such products and increase the likelihood that existing users of tobacco products will stop using such products.

## Additional files

Additional file 1: Table S1. Characteristics of included studies on menthol cigarettes and smoking initiation. Table including Reference, Study Design, Setting, Study Population, Sample Size, and Outcomes (DOCX 67 kb)

Additional file 2: Table S2. Characteristics of included studies on menthol cigarettes and nicotine dependence. Table including Reference, Study Design, Setting, Study Population, Sample Size, and Outcomes (DOCX 33 kb)

Additional file 3: Table S3. Characteristics of included studies on menthol cigarettes and smoking cessation. Table including Reference, Study Design, Setting, Study Population, Sample Size, and Outcomes (DOCX 57 kb)

## Abbreviations

FDA: Food and Drug Administration
MTF: Monitoring the Future
NHANES: National Health and Nutrition Examination Survey
NHIS: National Health Interview Survey
NSDUH: National Survey on Drug Use and Health
NYSCS: National Youth Smoking Cessation Survey
NYTS: National Youth Tobacco Survey
PATH: Population Assessment of Tobacco and Health
TPSAC: Tobacco Product Scientific Advisory Committee
TUS-CPS: Tobacco Use Supplement to the Current Population Survey
