# Supplementary material for: Menthol cigarettes and the public health standard: a systematic review
Source: BMC Public Health. 2017 Dec 29;17:983. doi: 10.1186/s12889-017-4987-z (PMC5747135; doi:10.1186/s12889-017-4987-z)
Supplement: Supplementary file 1 — Characteristics of included studies on menthol cigarettes and smoking initiation. Table including Reference, Study Design, Setting, Study Population, Sample Size, and Outcomes (DOCX 67 kb) [file 12889_2017_4987_MOESM1_ESM.docx]

**Table S1 Characteristics of included studies on menthol cigarettes and smoking initiation**

| **Reference** | **Study Design** | **Setting** | **Study Population** | **Sample Size** | **Outcomes** |
| --- | --- | --- | --- | --- | --- |
| Agaku (2015) [58] | Cross-sectional | Household survey  Eurobarometer 385 | Adults aged 15 years who were current or former cigarette smokers | 12,678 | Importance of cigarette design and marketing on initial smoking |
| Appleyard (2001) [26] | Cross-sectional | National, school-based survey  2000 NYTS | Youth who identify as  Asian, Native Hawaiian, or other Pacific Islander | 2,229 | Smoking prevalence |
| Azagba (2014) [47] | Cross-sectional | School-based survey in Canada, 2010-2011 | Canadian high school students grades 7-12 | Weighted sample characteristics (n=220,633 binge drinking; n=248,039 marijuana use) | Correlation of menthol cigarette use with binge drinking and marijuana use |
| Azagba (2014) [38] | Cross-sectional | School-based survey in Canada, 2010-2011 | Canadian high school students grades 9-12 who were current or experimental smokers | 4,736 | Smoking intensity and intent to continue smoking |
| Brennan (2015) [59] | Cross-sectional | Web-based, September - November 2012 | Youth aged 12-17 years and young adults aged 18-24 years | Youth  n = 553 current nonsmokers;  Young adult  n = 586 never tobacco users  n = 751 never tobacco users and current menthol cigarette user  n = 893 never tobacco users and current tobacco users | Menthol intention; Tobacco intention; Menthol behavior; Tobacco behavior |
| Caraballo (2011) [19] | Cross-sectional | 2004, 2006, 2009 NYTS  2004-2009 NSDUH  2003-2008 National Health and Nutrition Examination Survey (NHANES)  1998-2008 Monitoring the Future (MTF) | Nationally representative samples; ages vary | NYTS:  Middle school n = 1,978  High school  n = 6,163  NSDUH:  Youth n = 9,595  Adult n = 62,010  MTF:  8^th^ grade n =  20,863  10^th^ grade n =  30,722  12^th^ grade n = 40,914  NHANES:  n = 2,319 | Menthol use |
| Centers for Disease Control and Prevention (2009) [29] | Cross-sectional | National, school-based survey  2004 and 2006 NYTS | Youth aged 12-17 who were current smokers | Middle School  n = 26,257  High School  n = 28,044 | Brand preference |
| Connolly (2011) [57] | Cross-sectional | 1) 1996 and 2000 survey first reported in Oaki et al.  2) Japanese National Health and Nutrition Survey | Japanese junior and senior high school students; Randomly selected Japanese adults (20-69 years old) | Not reported | Menthol use |
| Curtin (2014) [64] | Cross-sectional | NSDUH; NHANES; NHIS; TUS-CPS | Nationally representative samples; ages vary | Not reported | Age of first cigarette smoked; first regular smoking; first daily smoking |
| Curtin (2014) [40] | Cross-sectional | NSDUH; NHANES; NHIS; TUS-CPS | Nationally representative samples; ages vary | Not reported | Past-month smoking;  regular smoking;  daily smoking |
| Dauphinee (2013) [37] | Longitudinal | School-based surveys | Youth in grades 6-9 enrolled in a California school district | For cross-sectional analysis:  n = 2,589  For longitudinal analysis:  n = 1,179 | Brand recognition |
| Delnevo (2015) [39] | Cross-sectional | National, phone-based survey  2011 National Young Adult Health Survey | Young adults (ages 18-34 years) who reported having smoked 100 cigarettes in their lifetime | 909 | Smoking frequency |
| Fallin (2015) [31] | Cross-sectional | National, phone-based survey  2009-2010 National Adult Tobacco Survey | Adult current smokers | 113,272 | Menthol use |
| Farrelly (2001) [24] | Cross-sectional | National, school-based survey  2000 NYTS | Middle school and high school students in grades 6-12 | 35,828 | Menthol use |
| Farrelly (2000) [23] | Cross-sectional | National, school-based survey  1999 NYTS | Middle school and high school students in grades 6-12 | 15,058 | Menthol use |
| Giovino (2015) [13] | Cross-sectional | National, household survey  2004-2010 NSDUH | U.S. civilian noninstitutionalized population aged 12 years or older | 389,698 | Menthol use |
| Hersey (2006) [34] | Cross-sectional | National, school-based survey  2000 and 2002 NYTS | Middle school and high school students in grades 6-12 | 2000 NYTS n=5,512  2002 NYTS n=3,202 | Menthol use |
| Hersey (2010) [28] | Cross-sectional | National, school-based survey  2006 NYTS | Middle school and high school students in grades 6-12 | 27,038 | Menthol use |
| Hickman (2014) [32] | Cross-sectional | National, household survey  2008-2009 NSDUH | U.S. civilian noninstitutionalized population aged 12 years or older | 24,157 | Menthol use |
| Klausner (2011) [52] | Content analysis | Searches in the Legacy Tobacco Documents Library between February and April 2010 | Internal tobacco industry documents | 128 | Industry perspective |
| Kong (2013) [48] | Cross-sectional | School-based survey in CT | Study 1: High school students at single high school  Study 2: Adolescent daily smokers participating in high school-based cessation intervention across multiple schools in New Haven County | Study 1, n=837 Study 2, n=132 | Menthol use |
| Kreslake (2008) [54] | Content analysis | Searches in the Legacy Tobacco Documents Library and Tobacco Documents Online of items dated from 1985 to 2007 | Internal tobacco industry documents | 580 | Industry perspective |
| Minaker (2014) [21] | Cross-sectional | School-based survey in Canada, 2010-2011 | Canadian high school students grades 9-12 who were past 30-day cigarette smokers | 5,035 | Correlates of menthol use |
| Moolchan (2004) [20] | Cross-sectional | Telephone survey, conducted between September 1999 and December 2002 | Baltimore-area teenage smokers calling to request cessation treatment | 622 | Menthol preference |
| Muilenburg (2008) [35] | Cross-sectional | School-based survey based on NYTS | Middle and high school students in 6 public institutions in a large southeastern metropolitan area | 2,068 | Menthol use |
| Nonnemaker (2013) [36] | Cohort | Conducted in 83 schools in seven communities and five states in the United States from 2000-2003 | Youth who participated in all three waves of the American Legacy Longitudinal Tobacco Use Reduction Study (ALLTURS), were younger than 17 at baseline, had initiated smoking during waves 1 or 2 of the study and were non-established smokers at initiation | 638 | Transition from non-established to established smoking (at least 100 cigarettes lifetime, plus smoked on 20-30 of the past 30 days) |
| Osaki (2006) [56] | Cross-sectional | School-based surveys in junior and senior high schools in Japan, 1996 and 2000 | Junior and senior high school students (grades 7-12) | 1996 n = 115,894  2000 n = 106,297 | Share of menthol brand varieties |
| Rath (2015) [42] | Longitudinal | Data from the Legacy Young Adult Cohort Study collected in July 2011, January 2012, and July 2012 | Young adult current smokers (ages 18-34 years) | 267 | Menthol use;  menthol brand switching |
| Rath (2016) [33] | Cross-sectional | Wave 3 of the Truth Initiative Young Adult Cohort conducted in July 2012 | Young adult current tobacco users (ages 18-34 years) | 1,037 | Menthol use |
| Roberts (2016) [44] | Cross-sectional | National, household survey  2012-2013 NSDUH | U.S. civilian noninstitutionalized population aged 12 years or older | 136,147 | Menthol use |
| Rock (2010) [18] | Cross-sectional | National, household survey  2004-2008 NSDUH | U.S. civilian noninstitutionalized population aged 12 years or older | 277,960 | Menthol use |
| Schauer (2017) [49] | Cross-sectional | National, household survey  2004-2008 NSDUH | U.S. civilian noninstitutionalized population aged 12 years or older | 51,500 | Relationship between menthol cigarette and substance use |
| Soulakova (2017) [22] | Cross-sectional | National, phone-based survey  2010-2011 TUS-CPS | Adult daily smokers | 19,961 | Menthol use |
| Sterling (2013) [45] | Cross-sectional | Online survey at 6 colleges in southeastern U.S., conducted in October 2010 | Random sample of 5000 students between the ages of 18-30 at each school | 4,388 | Relationship between small cigar and menthol cigarette use |
| Substance Abuse and Mental Health Services Administration (2009) [16] | Cross-sectional | National, household survey  NSDUH | U.S. civilian noninstitutionalized population aged 12 years or older | 2004 n = 67,760  2008 n = 68,736 | Menthol use;  menthol as starter product |
| Substance Abuse and Mental Health Services Administration (2011) | Cross-sectional | National, household survey  2004 – 2010 NSDUH | U.S. civilian noninstitutionalized population aged 12 years or older | Not reported | Menthol use; menthol as starter product |
| Villanti (2012) [41] | Cohort | 2003-2005 National Youth Smoking Cessation Survey | 16-24 year olds who had ever smoked 20 lifetime cigarettes and who had smoked at least once during the previous 30 days | 2003 n = 2,582  2005 n = 1,431 responded, of which 1,045 were still smoking | Menthol brand switching |
| Villanti (2013) [46] | Cross-sectional | Web-based survey | Nationally representative sample of young adults aged 18-34 years | n=4196; current tobacco users with complete data on flavored and menthol use (n=978) | Relationship between any flavored tobacco product use and menthol tobacco product use |
| Villanti (2016) [14] | Cross-sectional | National, household survey  2004 - 2014 NSDUH | U.S. civilian noninstitutionalized population aged 12 years or older who are current smokers | Not reported | Menthol use; relationship between menthol use and other tobacco product use |
| Villanti (2017) [15] | Cross-sectional | Wave 1 data from the PATH study collected in 2013-2014 | U.S. civilian noninstitutionalized population aged 12 years or older | 45,971 | Menthol use;  age of initiation |
| Vilsaint (2004) [25] | Cross-sectional | National, school-based survey  2002 NYTS | Students in grades 6-12 | 26,149 | Menthol use |
| Yerger (2011) [53] | Content analysis | Searches in the Legacy Tobacco Documents Library between February and April 2010 | Internal tobacco industry documents | 309 | Industry perspective |
| Yu (2011) [27] | Cross-sectional | National, school-based survey  2006 NYTS | American Indian and Alaska Native middle and high school students | n=305; weighted n=142,989 | Menthol use |

Note: Industry documents [50-51] and the 2012 report by Morgan Stanley [55] have been omitted from the above table
