# Supplementary material for: Menthol cigarettes and the public health standard: a systematic review
Source: BMC Public Health. 2017 Dec 29;17:983. doi: 10.1186/s12889-017-4987-z (PMC5747135; doi:10.1186/s12889-017-4987-z)
Supplement: Supplementary file 2 — Characteristics of included studies on menthol cigarettes and nicotine dependence. Table including Reference, Study Design, Setting, Study Population, Sample Size, and Outcomes (DOCX 33 kb) [file 12889_2017_4987_MOESM2_ESM.docx]

**Table S2 Characteristics of included studies on menthol cigarettes and nicotine dependence**

| **Reference** | **Study Design** | **Setting** | **Study Population** | **Sample Size** | **Outcomes** |
| --- | --- | --- | --- | --- | --- |
| Ahijevych (1999) [66] | Cross-sectional | Clinical research center | Women stratified by ethnicity and menthol/non-menthol preference recruited from community-based settings and worksites | 95 | Time to first cigarette |
| Collins (2006) [60] | Cross-sectional | Phone-based | Adolescent smokers recruited for a cessation treatment study | 572 | Time to first cigarette |
| Curtin (2014) [64] | Cross-sectional | National Survey on Drug Use and Health; NHANES; NHIS; TUS-CPS | Nationally representative samples; Ages vary across surveys | Not reported | Time to first cigarette; Cigarettes per Day |
| DiFranza (2004) [61] | Cohort | Individual interviews of students every 4 months between January 1998 and June 2000 | 7th grade cohort in two small cities in central MA who had ever inhaled a cigarette, prior to or during the study | 237 (120 who could recall mentholation of first inhaled cigarette) | Hooked on Nicotine Checklist |
| Fagan (2015) [6] | Cross-sectional | Translational research laboratory at the University of Hawaii Cancer Center | Adult daily smokers aged 18-35 years | 186 | Fagerstrom Test for Nicotine Dependence |
| Frost-Pineda (2014) [70] | Cross-sectional | Conducted at 39 clinical sites in the United States | Adult current smokers | 3,500 adults (1,100 menthol; 2,400 non-menthol) | Time to first cigarette; Fagerstrom Test for Nicotine Dependence |
| Hersey (2006) [34] | Cross-sectional | National, school-based survey  2000 and 2002 NYTS | Youth | 2000 NYTS n=5,512  2002 NYTS n=3,202 | Nicotine Dependence Scale for Adolescents |
| Hersey (2010) [28] | Cross-sectional | National, school-based survey 2006 NYTS | Middle and high school students in grades 6-12 | 27,038 | Time to first cigarette; Craving; Withdrawal symptoms |
| Kasza (2014) [69] | Cohort | Telephone interviews, conducted between 2002-2011 | Adult current smokers | 5,932 | Time to first cigarette; Cigarettes per day |
| Li (2012) [63] | Cross-sectional | School-based survey  2006-2009 subset of the National Year 10 Action on Smoking and Health (ASH) Snapshot Survey | New Zealand Year 10 students age 14-15 years who reported smoking at least 1-10 cigarettes in their lifetime and currently smoking at least on a monthly basis | 13,458 | Hooked on Nicotine Checklist |
| Muscat (2012) [67] | Cross-sectional | Community based health promotion and education program in Mt. Vernon, NY, 1990-2001 | Adult current daily cigarette smokers who had smoked at least 5 CPD for one or more years | 495 | Time to first cigarette |
| Nonnemaker (2013) [36] | Cohort | Conducted in 83 schools in seven communities and five states in the United States from 2000-2003 | Youth who participated in all three waves of the American Legacy Longitudinal Tobacco Use Reduction Study (ALLTURS), were younger than 17 at baseline, had initiated smoking during waves 1 or 2 of the study and were non-established smokers at initiation | 638 | Time to first cigarette; Craving |
| Rosenbloom (2012) [68] | Cross-sectional | Telephone screening questionnaire for RCT | Female smokers interested in participating in a RCT involving exercise and NRT (nicotine patch) in Boston, MA | 928 | Time to first cigarette; Cigarettes per day |
| Wackowski (2007) [62] | Cross-sectional | National, school-based survey  2004 NYTS | High school students in grades 9-12 who were current smokers | 2,597 | Time to first cigarette; Craving; Withdrawal symptoms |
