# Supplementary material for: Menthol cigarettes and the public health standard: a systematic review
Source: BMC Public Health. 2017 Dec 29;17:983. doi: 10.1186/s12889-017-4987-z (PMC5747135; doi:10.1186/s12889-017-4987-z)
Supplement: Supplementary file 3 — Characteristics of included studies on menthol cigarettes and smoking cessation. Table including Reference, Study Design, Setting, Study Population, Sample Size, and Outcomes (DOCX 57 kb) [file 12889_2017_4987_MOESM3_ESM.docx]

**Table S3 Characteristics of included studies on menthol cigarettes and smoking cessation**

| **Reference** | **Study Design** | **Setting** | **Study Population** | **Sample Size** | **Outcomes** |
| --- | --- | --- | --- | --- | --- |
| Alexander (2010) [72] | Cross-sectional | 2006-07 TUS CPS | Adults current Smokers | 30,176 | Quit attempts in past year |
| Blot (2011) [92] | Cohort | Southern Community Cohort Study, prospective cohort in 12 southern states from 2002-2009 | Ever smoking adults aged 40-79 years | 12,373 | Quit rates at enrollment |
| Cubbin (2010) [81] | Cross-sectional | 2005 National Health Interview Survey and Cancer Control Supplement | Adults aged 25-64 years who self-identified as (1) Black, non-Hispanic/Latino; (2) Hispanic/Latino; or (3) White, non-Hispanic | Current every day smokers n=3902; Former smokers n=3786 | Quit attempts in past year; Duration of abstinence among former smokers |
| Delnevo (2011) [76] | Cross-sectional | 2003 & 2006/2007 TUS-CPS | White, black and Hispanic ever smokers (current smokers; former smokers who quit in past 5 years) | Pooled data from 2003 and 2006/7  Set 1: 71,193  Set 2: 65,316  Set 3: 55,322  Set 4: 50,761  Set 5: 24,465 | Quit rate |
| D'Silva (2012) [88] | Cohort | Telephone and mail surveys of ClearWay Minnesota conducted between 2009-2011; Follow-up at 7 months | Random sample of cigarette smokers who called the quitline, registered for counseling services, and responded to a question about menthol cigarettes | 715 with baseline and follow-up data | 30-day PPA, ITT; Predictors of 30-day PPA |
| Fagan (2007) [73] | Cross-sectional | 2003 TUS-CPS | Young adult smokers aged 18-30 years | 7,912 | Quit attempts in past year |
| Fagan (2010) [78] | Cross-sectional | 2003 and 2006-07 TUS-CPS | Adults daily smokers | 46,273 | Quit attempts in past year; Duration of abstinence |
| Faseru (2013) [99] | Randomized controlled trial | Community-based | African American light smokers (10 or fewer cpd) | 540 | 7-day PPA |
| Fu (2008) [96] | Randomized controlled trial | VA medical centers | Aged 19+ and who had received a prescription for NRT or bupropion | 1,343 (menthol n=342; non-menthol n=1,001) | 7-day PPA |
| Gandhi (2009) [91] | Cohort | Specialized smoking cessation outpatient clinic in New Jersey | Patients who set a quit date and attempted to quit smoking, between 1 January 2001 and 30 June 2005 | 1,688 (menthol n=778; non-menthol n=910) | 7-day PPA at 4 weeks and 6 months |
| Gundersen (2009) [82] | Cross-sectional | 2005 National Health Interview Survey - Cancer Control Supplement; Conducted via computer-assisted personal interview | White, black and Hispanic current and former smokers who had ever attempted to quit and do not use other tobacco products | 7815 | Quit rate |
| Hyland (2002) [89] | Cohort | COMMIT Trial | Baseline smokers who completed a telephone survey in 1988 and follow-up interview in 1993 | 13,268 (3,184 Menthol; 10,084 Non-Menthol) | 6-month abstinence |
| Kahende (2011) [74] | Cross-sectional | 2003 TUS-CPS | Adults who have smoked in the past year (former or current who smoked reported smoking at least 100 cigarettes during lifetime and smoked within past year) | 16,213 | Quit attempts |
| Keeler (2016) [79] | Cross-sectional | 2006/07 and 2010/11 TUS-CPS | Adults who quit smoking within the past 12 months | 54,448 | Quit intention; quit attempts; quit rate |
| Levy (2011) [75] | Cross-sectional | 2003 and 2006/07 TUS-CPS | Current and former smokers | 2003 Wave, n=34,260  2007 Wave, n=31,250 | Quit attempts in past year; Duration of abstinence |
| Lewis (2014) [94] | Cohort | Nielsen Homescan Panel | Adults | 18,103 | Quit Rate |
| Muscat (2002) [84] | Cross-sectional | Hospital-based case-control study of tobacco related cancers; Conducted in New York, PA, and DC between 1981-1999 | Current and former smokers | 19,545 (15.4% menthol smokers; defined as last brand of cigarette smoked) | Rate of former smoking |
| Okuyemi (2003) [97] | Randomized controlled trial | Community-based health care center, 1999-2000 | African American adult smokers | 600 (471 menthol, 129 non-menthol) | 7-day PPA at 6 weeks and 6 months |
| Okuyemi (2004) [85] | Cross-sectional | Inner-city health center | Adults current smokers who were inner-city residents | 480 | Recency of quit attempts; Most recent duration of abstinence; Longest ever duration of abstinence |
| Okuyemi (2007)[98] | Randomized controlled trial | Community-based health center serving a predominantly African American population | African Americans light smokers (<=10 cpd) | 755 (615 menthol, 140 non-menthol) | 7-day PPA at 8 and 26 weeks |
| Pletcher (2006) [90] | Cohort | Multi-center U.S. cohort study (CARDIA) | African American and European American aged 18-30 years and healthy at the time of enrollment in 1985 | 1,544 (972 Menthol, 563 Non-Menthol) | Successful smoking cessation if recent quit attempt made |
| Rath (2015) [42] | Cohort | Legacy Young Adult Cohort Study | Adults aged 18-34 years who either remained current cigarette smokers across all three time points of study (n=212) or initiated cigarette smoking at Time 2 and remained cigarette smokers at Time 3 (n=58) | 267 | Quit intention |
| Reitzel (2011) [87] | Cohort | Re-analysis of RCT in Houston, TX between Oct 2004 and April 2008 evaluating the efficacy of a Motivation and Problem Solving (MAPS) treatment for the prevention of postpartum relapse - adjusted for treatment group | Population Adults women at 30-33 weeks of pregnancy at enrollment who reported smoking ≥ 1 cigarette daily for year prior to pregnancy and stopped during pregnancy or within 2 months prior to becoming pregnant | 244 (123 Menthol, 121 Non-Menthol) | Continuous abstinence from smoking 26 weeks postpartum |
| Reitzel (2013) [93] | Cohort | "Community smokers attempting to quit." Participants were smokers from Houston, TX | Non-Hispanic White and Non-Hispanic Black adult daily smokers | 183 | Continuous short-term abstinence |
| Reitzel (2013) [86] | Cross-sectional survey (Part of a lung cancer case-control study) | Parent study conducted at The University of Texas MD Anderson Cancer Center; Questionnaire administered between February 1996 and July 2011 | Non-Hispanic White and Non-Hispanic Black adults who endorsed smoking at least 100 cigarettes in their lifetimes and smoking within the last year | 1067 | Quit Intention |
| Rojewski (2014) [101] | Randomized controlled trial | Secondary analysis of data from an RCT of low-dose naltrexone augmentation of nicotine replacement | Weight-concerned smokers | 166 (61 Menthol, 105 Non-Menthol) | 7-day PPA at 26 weeks |
| Rosenbloom (2012) [68] | Cross-sectional | Telephone screening questionnaire for RCT | Female smokers interested in participating in a RCT involving exercise and NRT (nicotine patch) in Boston, MA | 928 | Quit duration |
| Smith (2014) [102] | Randomized controlled trial | Two communities in Wisconsin, USA | Adults who smoked at least 10 cpd during the past 6 months and reported being motivated to quit smoking | 1,504 | 7-day PPA at 4, 8, and 26 weeks |
| Stahre (2010) [80] | Cross-sectional | 2005 National Health Interview Survey Cancer Control Supplement | Adults smokers with known menthol smoking status | 12,004 (Current smokers - 1700 menthol, 4355 non-menthol, 456 no usual type; Former smokers - 1515 menthol, 4434 non-menthol, 825 no usual type) | Quit attempts in past year  Population quit ratio |
| Sulsky (2014) [83] | Cross-sectional | NHIS 2005 and 2010; TUS-CPS 2010/2011 | Adult current and former smokers | Not reported | Duration of abstinence |
| Trinidad (2010) [77] | Cross-sectional | 2003 and 2006-07 TUS-CPS | Current and former smokers aged 20-65 years | 125,639 (69.8% of African American smokers used mentholated cigarettes vs 20-25% in other racial/ethnic groups) | Quit intention; Cessation of at least 6 months |
| Winhusen (2013) [100] | Randomized controlled trial | Conducted at 12 SUD outpatient treatment programs that did not provide smoking cessation treatment as part of their standard treatment | Adults who met DSM-IV-TR criteria for cocaine- and/or methamphetamine-dependence, were enrolled in outpatient SUD treatment, smoked at least 7 cpd and had a CO level >=8ppm | 510 (301 cocaine-dependent, 209 methamphetamine-dependent) | Quit rate |
